# Physical health-related quality of life predicts disability pension due to musculoskeletal disorders: seven years follow-up of the Hordaland Health Study Cohort

**DOI:** 10.1186/1471-2458-14-167

**Published:** 2014-02-14

**Authors:** Inger Haukenes, Erlend H Farbu, Trond Riise, Grethe S Tell

**Affiliations:** 1 Department of Global Public Health and Primary Care, University of Bergen, Kalfarveien 31, Bergen NO-5018, Norway; 2 Department of Public Mental Health, Division of Mental Health, Norwegian Institute of Public Health, Bergen, Norway

**Keywords:** Cohort study, Disability pension, Physical health, Musculoskeletal disorders, Mental health, Quality of life, Self-reported health

## Abstract

**Background:**

Musculoskeletal diseases are characterized by a high degree of comorbidity with common mental disorders and are a major cause of health-related exclusion from working life. Using a prospective design we aimed to examine the relative importance of physical and mental health-related quality of life as predictors of disability pension due to musculoskeletal diseases.

**Methods:**

A subsample (N = 18581) born 1953–1957, participated in the The Hordaland Health Study (HUSK) during 1997–1999, and was followed through December 31^st^ 2004. Baseline measures of health-related quality of life were estimated using the Physical (PCS) and Mental Component Summary (MCS) of the Short Form-12 (SF-12). Further information on education, occupation, smoking, physical activity, number of musculoskeletal pain sites and BMI were provided by questionnaires and health examination. The association between self-perceived physical and mental health and subsequent disability pension, obtained from the national database of health and social benefits was estimated using Cox regression analyses.

**Results:**

Participants reporting poor physical health (quartile 1) had a marked increased risk for disability pension due to musculoskeletal diseases (age and gender-adjusted hazard ratio = 22.1, 95% CI = 12.5–39.0) compared with those reporting good/somewhat good physical health (quartiles 4 and 3 combined). Adjustment for socioeconomic status and lifestyle factors slightly attenuated the association (hazard ratio = 16.7), and adding number of reported pain sites weakened the association even more (hazard ratio = 7.1, 95% CI = 3.8–12.8). Also, participants reporting poor mental health had a higher risk for disability pension due to musculoskeletal diseases (age and gender adjusted hazard ratio = 1.8, 95% CI = 1.3–2.6); however, in the final model the risk was not statistically significant.

**Conclusions:**

The physical component in health-related quality of life (SF-12) was a strong predictor of disability pension due to musculoskeletal diseases, whereas the mental component played a less prominent role.

## Background

Musculoskeletal diseases have substantial impact on individuals’ quality of life and are a common cause of sickness absence and disability pension in countries with universal welfare schemes [1].

Studies have found a strong association between self-rated health, measured with a single global health question, and future health outcomes like sickness absence [2,3] disability pension and all-cause mortality [2,4,5].

Health-related quality of life (HRQOL) measures the individual’s perception of current health status and its impact on essential spheres in life, such as ability to carry out ordinary work and domestic tasks. HRQOL can be divided into self-perceived physical health and self-perceived mental health [6]. Several studies have found that self-perceived physical health is a strong predictor of mortality [7,8]. Moreover, a population-based study found that self-perceived physical health was strongly associated with subsequent sickness absence, whereas the impact of self-perceived mental health was substantially weaker [9]. However, there is a lack of studies examining the predictive ability of self-perceived physical and mental health (HRQOL) on disability pension.

In Nordic countries approximately one third of the disability diagnoses are linked to musculoskeletal disorders and one third to common mental disorders [10]. Musculoskeletal diseases are characterized by a high degree of comorbidity with common mental disorders [11]. A longitudinal study found that widespread pain was a strong predictor of disability pension due to musculoskeletal diseases (MSD) but did also predict disability pension due to mental diseases [12].

Some have suggested that mental health is under recognised as a cause of disability pension on the expense of physical diagnoses, such as musculoskeletal diseases [13,14]. Consequently, strategies aiming at preventing reduced work ability due to MSD may overestimate the importance of physical and ergonomic risk-factors and disregard factors influencing mental health.

Thus, the aim of the current study was to examine the relative importance of self-perceived physical and mental health in HRQOL as predictors of subsequent disability pension due to musculoskeletal diseases, in a middle-aged working population.

## Methods

### Population and data material

The Hordaland Health Study (HUSK) was conducted during 1997–99 as a collaboration between the National Health Screening Service, the University of Bergen and local health services. The source population included all individuals in Hordaland county born 1953–57 (29,400). A total of 8,598 men and 9,983 women participated, yielding a participation rate of 57% for men and 70% for women.

Baseline measurements included height and weight and self-administered questionnaires provided information on HRQOL, physical activity, smoking, alcohol consumption, occupation and musculoskeletal pain.

In the current study we included only participants who at baseline reported at least 100 hours paid work during the last 12 months, excluding thus 1784 participants. Moreover we excluded individuals with all-cause DP at baseline (time = 0), and the following 12 months (n = 50). The final study population included 7934 men and 8488 women aged 40–46 years at baseline.

### Predictor variable

HRQOL was assessed using the Short-Form 12 (SF-12) questionnaire, a shorter version of SF-36 [6]. The SF-12 includes 12 items measuring health status and limitations in daily life due to physical and mental health problems. These 12 questions may be accumulated in to 8 subscales which again are summed into a physical component summary score (PCS) and a mental component summary score (MCS). And although all 8 scales are included in the calculation of both the PCS and MCS, the scales that are given the highest weights in the PCS score are the 4 “physical health scales”; physical functioning, role limitation due to physical problems, pain and general health. Similarly the scales given the highest weights in the MCS score are the 4 “mental health scales”; social functioning, role limitations due to emotional problems, mental health and energy/vitality. The PCS and MCS were standardized according to the general population with a mean score of 50 (SD 10) [6]. Lower scores indicate poor health and higher scores good health. The developers of this instrument used a factor analysis with an orthogonal rotation for generating the factor loadings for PCS and MCS [6]. This means that the two measures are structurally independent of each other.

The PCS and MCS were in this study divided into quartiles and missing values from one or more items were categorised in a separate group and included in the analyses. The cut-off values for quartiles on PCS and MCS were similar for men and women (PCS, quartile 1 = 49.0, quartile 2 = 53.4, quartile 3 = 55.3 and for MCS, quartile 1 = 47.8, quartile 2 = 53.2 and quartile 3 = 55.9).

### Outcome

In Norway, disability pension is a universal right for individuals between 18 and 67 years of age, whose earning capacity has been permanently or at least 50% reduced due to long-term sickness, injury or disability. An application for disability pension can only be considered after 12 months sick leave, appropriate medical treatment and rehabilitation, or training for other kind of work. The disability diagnosis is the primary diagnosis, i.e. the medical condition that most critically affects the earning capacity.

The main study outcome was award of disability pension (50% or more) due to MSD in the period from one year after participation in HUSK until the end of follow-up on December 31^st^ 2004. Events of disability pension were obtained from “FD-trygd”, a national database of health and social benefits. The data linkage between HUSK and FD-trygd was made possible by the personal identification number unique to each resident in Norway. The main outcome of the current study is defined by ICD9 codes ranging from 710 through 739 and ICD10 codes starting with an M, according to the codes of the year the disability pension was registered.

### Baseline covariates

Heavy physical activity (causing sweating and heavy breathing) was reported in four categories “0 hours”, “less than 1 hour”, “1-2 hours “or “3 hours or more” per week the last year. Participants were categorized into “non-smokers”, “former smokers” or “smokers”. Height and weight were measured by health professionals according to standard procedure and used to estimate Body Mass Index (BMI, kg/m^2^). BMI was classified according to WHO’s criteria: underweight <18.5, normal weight 18.5-24.9, overweight 25–30 and obese >30.

Level of education was reported in six categories, elementary school (< 7 years), lower secondary school (7–9 years), vocational school (10–12 years), higher secondary school (12 years), university/university college (<4 years) and university/university college (≥4 years). The two first categories were collapsed into one category, primary school (grades 1–9).

Self-reported occupation and branch of industry were coded according to the International Standard Classification of Occupations, ISCO-88, after which an international applicable algorithm by Ganzeboom and Treiman was used to classify ISCO-88-codes into Erikson, Goldthorp and Portocareros social class scheme (EGP-scheme) [15,16]. The following five categories were used: administrative and professionals, routine non-manual, skilled manual, unskilled manual and primary industry (agriculture). Studies have found that the EGP-scheme reflects the risk associated with disability pension [17].

Musculoskeletal pain sites were assessed by the question: “Have you during the last year had pain and/or stiffness in muscles or joints lasting more than 3 months?” If yes, the participants indicated the location(s): neck, shoulder, elbow, hands/wrist, chest/abdomen, upper back, lower back, hips, knees, ankles or feet. Possible locations for pain sites were summed up and grouped into 0, 1, 2 and ≥3 pain sites [12].

### Statistical analyses

Mean scores and 95% confidence intervals (95% CI) of PSC and MCS across strata of predictor variable and covariates were calculated. We used Cox proportional hazard analyses with hazard ratio (HR) and 95% CI to estimate age and gender-adjusted associations between the predictor variable and outcome and covariates and outcome. Due to few disability events (MSD) among participants reporting good physical health (PCS, quartile 4), we combined quartiles 4 and 3 and used this category as a reference group. This was also done for MCS, although the distribution of disability events (MSD) across quartiles was more even. For the covariates the reference groups were high socioeconomic status, never smokers, physically active, normal BMI and zero pain sites.

The association between quartiles of PCS and risk for disability pension due to MSD was also illustrated using Kaplan Meyer survival curves. Moreover, we examined potential interaction between PCS and gender and MCS and gender in the Cox regression analyses. Finally, we evaluated the proportional hazard assumption by inspecting the log minus log plots stratified on the level for each covariate and found no major deviation from a proportional hazard.

Multivariate models were used to examine the association between quartiles of self-reported health (MCS and PCS) and disability pension due to MSD. In similar analyses we also examined the relation between PCS and MCS and disability pension due to mental diagnoses. The first model was adjusted for age, gender, education, EGP-scheme, smoking, physical activity and BMI. In the second model we added the number of pain sites. The endpoint was defined as date for award of disability pension due to MSD. Participants were otherwise censored at date of death, date of emigration, date of award of disability pension due to diagnoses other than MSD or end of follow-up, whichever occurred first.

Since the risk factors for disability pension due to MSD might also be related to the risk for disability pensioning due to mental diagnoses and other diagnoses and even death, we also performed Cox regression analyses using disability pensioning due to these events as competing risk outcomes. These analyses were performed in STATA using the Fine and Grey approach [18].

The PCS and MCS are made uncorrelated due to the orthogonal factor rotation in the construct of these variables. Therefore, in order to test whether mental health influenced the association between PCS and DP due to MSD, we also adjusted for the sub-scale mental health in SF-12. This subscale is not uncorrelated to the PCS. We also performed additional analyses with 2 years wash-out period for disability events, to examine the risk of misclassification by participants applying for disability pension while reporting their health status in HUSK.

The analyses were conducted using IBM SPSS 18.

### Ethical approval

The study protocol was approved by the Regional Committee for Medical Research Ethics, Western Norway and by the Norwegian Data Inspectorate. Written statements of informed consent were obtained from all participants at baseline.

## Results

During follow-up a total of 214 (1.3%) individuals were awarded disability pension due to MSD.

Smokers, physically inactive, obese, lower educated and unskilled manual workers (EGP) had higher risk for disability pension due to MSD, compared with their reference groups (Table 1). Individuals with higher education or classified in higher occupational classes perceived their health as better than those with lower ranks, whereas a similar trend was not present for mental health. Smoking and physical inactivity were associated with poor self-perceived physical and mental health. Higher BMI was only associated with poorer physical health. No participants were underweight.

**Table 1:** **Mean (95**% **CI) values of self-perceived physical and mental health and risk for disability pension due to musculoskeletal disorders across baseline characteristics**

							**Disability pension**			
	**Total**		**PCS**		**MCS**				**Adjusted for gender and age**	
	**N**	**%**	**Mean**	**95% CI**	**Mean**	**95% CI**	**N**	**%**	**HR**	**95% CI**
HRQOL*, PCS^**†**^										
Quartiles 3 and 4 (good health)	7357	44.8	55.6	55.6–55.7	50.3	50.1–50.5	13	0.2	1.0	
Quartile 2	3737	22.8	51.6	51.5–51.6	51.3	51.1–51.6	21	0.6	3.3	1.7– 6.6
Quartile 1 (poor health)	3697	22.5	40.5	40.3–40.8	49.3	49.0–49.6	150	4.1	22.1	12.5–39.0
Missing	1631	9.9					30	1.8	9.7	5.0–18.5
HRQOL*, MCS^**‡**^										
Quartiles 3 and 4 (good health)	7162	43.6	51.0	50.9–51.2	56.3	56.3–56.4	69	1.0	1.0	
Quartile 2	3953	24.1	50.9	50.6–51.1	50.9	50.8–50.9	44	1.1	1.1	0.8–1.6
Quartile 1 (poor health)	3676	22.4	50.4	50.2–50.7	38.0	37.8–38.3	71	1.9	1.8	1.3–2.6
Missing	1631	9.9					30	1.8	1.7	1.1–2.6
**Gender**										
Men	7934	48.3	51.3	51.1–51.4	51.0	50.8–51.2	47	0.6	1.0	
Women	8488	51.7	50.4	50.2–50.6	49.6	49.4–49.8	167	2.0	3.3	2.4–4.6
**Education**										
University/university college ≥ 4 y.	2837	17.4	52.1	51.9–52.4	49.8	49.5–50.1	10	0.4	1.0	
University/university college < 4 y.	3202	19.6	51.7	51.5–51.9	50.0	49.7–50.3	19	0.6	1.7	0.8–3.6
Secondary high school	1638	10.0	51.1	50.8–51.5	50.1	49.7–50.5	18	1.1	3.0	1.4–6.5
Vocational school	5954	36.5	50.5	50.3–50.7	50.9	50.6–51.1	92	1.5	4.3	2.2–8.2
Primary school (Grades 1–9)	2686	16.5	49.0	48.6–49.3	50.1	49.7–50.5	73	2.7	7.1	3.7–13.8
**Occupational class**										
Administrative and professionals	5527	33.7	51.9	51.7–52.0	50.5	50.3–50.7	27	0.5	1.0	
Routine non-manual	5295	32.3	50.9	50.7–51.1	49.8	49.5–50.0	69	1.3	1.7	1.1–2.7
Skilled manual	3579	21.8	50.2	49.9–50.4	50.8	50.5–51.0	63	1.8	3.2	2.0–5.0
Unskilled manual	1433	8.7	48.3	47.9–48.8	50.2	49.7–50.7	49	3.4	5.7	3.5–9.1
Primary industry	576	3.5	50.6	50.0–51.2	50.5	49.7–51.2	6	1.0	2.2	0.9–5.3
**Smoking**										
Never smoker	6103	37.2	51.3	51.2–51.5	50.8	50.6–51.0	51	0.8	1.0	
Former smoker	4555	27.8	51.1	50.9–51.3	50.7	50.5–51.0	46	1.0	1.2	0.8–1.8
Current smoker	5721	34.9	50.1	50.0–50.3	49.4	49.2–49.7	117	2.0	2.5	1.8– 3.4
**Physical activity per week**										
≥ 3 hours	2261	13.8	52.2	51.9–52.5	51.6	51.2–51.9	21	0.9	1.0	
1–2 hours	4700	28.6	51.5	51.3–51.7	50.8	50.5–51.0	31	0.7	0.6	0.4–1.1
< 1 hour	4515	27.5	50.9	50.7–51.1	50.0	49.7–50.3	47	1.0	1.0	0.6–1.7
Zero hours	4946	30.1	49.4	49.2–49.7	49.5	49.3–49.8	115	2.3	2.0	1.3–3.2
**BMI**										
Normal weight 18,5–24.9	8434	51.4	51.3	51.1–51.4	50.0	49.8–50.1	112	1.3	1.0	
Overweight 25-30	6260	38.2	50.8	50.6–51.0	50.7	50.5–50.9	63	1.0	1.0	0.7–1.3
Obese >30	1705	10.4	48.8	48.4–49.2	50.5	50.1–51.0	36	2.1	1.9	1.3–2.8
**Musculoskeletal pain sites**										
0	9434	57.4	53.6	53.5–53.6	51.3	51.1–51.5	21	0.2	1.0	
1	1571	9.6	50.4	50.0–50.7	50.8	50.3–51.2	19	1.2	5.6	3.0–10.4
2	1587	9.7	48.9	48.5–49.3	49.8	49.3–50.3	17	1.1	4.6	2.4–8.8
≥3	3830	23.3	45.0	44.7–45.3	47.8	47.5–48.2	157	4.1	16.7	10.6–26.4

*Health-related quality of life.

^†^Physical Component Summary.

^‡^Mental Component Summary.

### Physical component summary (PCS)

The proportion of granted disability pensions due to MSD increased with lower scores on PCS, with 150 disability events in quartile 1 (poorest physical health), 21 events in quartile 2, 10 events in quartile 3 and 3 events in quartile 4 (best health) (Table 1, quartiles 3 and 4 are merged).

**Figure 1:**
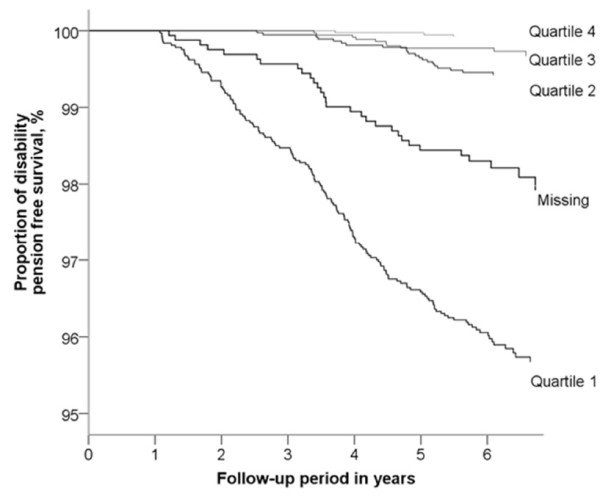
Proportion who were not awarded disability pension by quartiles of the Physical Component Summary (PCS) of the Short Form-12 (SF-12).

Individuals with the poorest physical health (PCS quartile 1) had approximately 22 times higher risk for subsequent disability pension due to MSD compared with participants reporting good and somewhat good physical health (PCS quartiles 3 and 4) (Table 1, Figure 1). Moreover, the risk was 3 times higher among those in the second quartile of PCS.

In multivariate analyses of the association between quartiles of PCS and disability pension due to MSD, adjusting for education, occupational class, smoking, physical activity and BMI attenuated the association; nevertheless, participants perceiving their physical health as poor (quartile 1) still had significantly higher disability risk (HR = 16.7) than the reference group (Table 2). Additional adjustment for musculoskeletal pain sites (model 2) further attenuated the association between PCS and disability pension; however, the disability risk in quartile 1 (HR = 7.1) remained significantly increased compared to the reference group (quartiles 3 and 4). Interaction between PCS and gender was not significant (p = 0.270).

**Table 2:** Association between self-perceived physical (PCS) and mental (MCS) health, and disability pension due to musculoskeletal diagnosis and mental diagnoses in two models of adjustments

	**Disability pension due to musculoskeletal diagnosis**		**Disability pension due to mental diagnosis**	
	Model 1^*^	**Model 2**^**†**^	Model 1*	**Model 2**^**†**^
	**HR (95% CI)**	**HR (95% CI)**	**HR (95% CI)**	**HR (95% CI)**
**HRQOL, PCS**				
Quartiles 4 and 3 (good health)	1	1	1	1
Quartile 2	2.8 (1.4–5.6)	1.9 (0.9–3.7)	1.4 (0.7–2.7)	1.2 (0.6–2.3)
Quartile 1 (poor health)	16.7 (9.5–29.7)	7.1 (3.8–12.8)	3.4 (1.9–5.9)	2.1 (1.1–3.9)
Missing	7.6 (3.9–14.6)	4.7 (2.4–9.2)	3.1 (1.5–6.0)	2.4 (1.2–4.9)
**HRQOL, MCS**				
Quartiles 4 and 3 (good health)	1	1	1	1
Quartile 2	1.2 (0.9–1.8)	1.0 (0.7–1.5)	1.3 (0.5–3.5)	1.3 (0.5–3.4)
Quartile 1 (poor health)	1.9 (1.3–2.6)	1.2 (0.9–1.7)	11.5 (5.9–22.4)	9.6 (4.9–18.9)
Missing	1.5 (1.0–2.4)	1.2 (0.8–1.9)	6.4 (2.9–14.1)	5.9 (2.7–13.1)

*Model 1. Adjusted for gender, education, occupational class, smoking, physical activity and BMI.

^†^Model 2. Adjusted for model 1+ musculoskeletal pain sites.

Using disability pensioning due to mental diagnoses, other diagnosis and death as competing risk outcomes rather than censoring these events, gave only marginally different estimates (data not shown).

Additional analyses with two years wash-out period only caused minor changes in the risk estimates (data not shown). Moreover, sensitivity testing with the sub-scale mental health (SF-12) did not change the estimates (data not shown).

Among the participants with missing values in PCS, more than 50% had missing on only one item. As a group, individuals with missing values had a 10-fold increased risk of disability pension due to MSD compared with the reference group (quartiles 3 and 4) (Table 1). Multivariate adjustments attenuated the hazard ratios; however, the persisting disability risk among missing was substantial (model 1, HR = 7.6, and model 2, HR = 4.7) (Table 2).

### Mental component summary (MCS)

Disability pension events were distributed more evenly across quartiles of MCS than quartiles of PCS (Table 1). The age and gender-adjusted disability risk due to MSD in quartile 2 was not significantly higher than the reference group (quartiles 3 and 4), whereas participants reporting the poorest mental health had 1.8 times higher disability risk (HR = 1.8, 95% CI 1.3–2.6) (Table 1). However, no significant difference persisted across quartiles of self-perceived mental health after multivariate adjustment for education, occupational class, smoking, physical activity, BMI and musculoskeletal pain sites (Table 2). Interaction between MCS and gender was not significant (p = 0.095).

### Disability pension due to mental diagnoses

Among the participants 93 individuals were granted disability pension due to mental diagnoses and among them 42 (1.1%) reported poor physical health (PCS quartile 1). In the multivariate cox regression analyses the disability risk associated with poor physical health was 2.1 times higher than those reporting good physical health (Table 2, model 2). Participants reporting poor mental health (MCS quartile 1) had 9.6 times higher risk for disability pension due to mental diagnoses compared with those reporting good mental health (Table 2, model 2).

## Discussion

### Main findings

In a 5 to 7 years prospective study of middle-aged men and women in Norway, poorer self-perceived physical health (SF-12) strongly predicted disability pension due to MSD, whereas poorer self-perceived mental health had little predictive value. Adjustment for lifestyle, socioeconomic factors and pain sites attenuated the disability risk associated with poorer self-perceived physical health; however, a strong association persisted after adjustments.

### Self-perceived physical health (PCS)

Previous studies have found an association between self-rated health measured with a single global health question and all-cause disability pension [5,19,20], disability pension due to back pain and long term sickness absence [3,21]. The association between sickness absence and self-rated health seems to be influenced by the state of being absent from work, as well as the medical reason for the absence [22]. Disability pension due to musculoskeletal diseases is usually preceded by repeated spells of sickness absence; thus, the predictive ability of self-rated health to foresee disability pension may be a reflection of the individual’s sickness absence history combined with increasingly reduced work ability due to health.

How a person perceives his or her health is not only an important factor in the assessment of work ability, it is also associated with the development and recovery from chronic musculoskeletal pain [23,24]. SF-12 contains a question that examines the impact of pain on daily life activities (work and leisure time); however, we do not know if this pain origins from the musculoskeletal apparatus, other organs or due to social or mental burdens. Thus, adjusting for musculoskeletal pain sites made it possible to examine the predictive ability of PCS in relation to disability pension due to MSD, taking account of musculoskeletal pain sites. The finding that the association between PCS and disability pension due to MSD remained strong after adjustment for number of pain sites, support findings that point to self-perceived physical health as a measure that incorporate health complaints other than musculoskeletal pain [12]. Consequently, additional health measures might have explained more of the association between PCS and subsequent disability pension due to MSD.

### Self-perceived mental health (MCS)

In the current study the limited predictive value of MCS was surprising. Literature point to a relationship between poor mental health and subsequent all-cause disability pension and disability pension not related to mental diagnosis [13]. Findings from the current study indicate that the relative importance of self-perceived mental health in the development of work-disability due to MSD is limited among middle-aged men and women. However, the orthogonal rotation used in SF-12 constructs PCS and MCS makes them uncorrelated, meaning that MCS only measure the variation in mental health that is not related to physical health (PCS) (and vice versa) [6]. Thus, a more explicit formulation of our finding would be that the independent influence of mental health on disability pension due to MSD was limited among middle-aged men and women.

However, the limited association between MCS and disability events due to MSD, does not rule out psychological processes in the development of MSD, work disability and self-perceived health. Studies have found that fear-avoidance beliefs and pain-catastrophizing are strongly associated with perception of musculoskeletal pain and disability pension due to low back pain [25]. Individuals that are less influenced by fear-avoidance beliefs may be less affected by their musculoskeletal pain and consequently perceive their health as relatively good. In the current study this may explain why musculoskeletal pain sites did not attenuate more of the association between self-perceived physical health and subsequent disability pension.

### Self-perceived health and early life-experiences

Moreover, early life experiences may influence perception of health and subsequent inflow to disability pension. However, Singh-Manoux et al. found that self-rated health mainly reflected current physical and mental health status and not early life factors, family history, socio-demographic variables, psychosocial factors or health behaviors [26]. Interpreted in the light of the present study the association between self-perceived health and disability pension due to MSD is most likely mediated through *current* restrictions in physical health rather than mental health. Similarly, *current* restrictions in mental health rather than physical health may mediate the association between poor self-perceived health and disability pension due to mental diagnoses.

### Self-perceived health and socioeconomic status

It is well acknowledged that disability pension is distributed according to a social gradient, with a higher risk among individuals with lower education and manual work [27]. A similar gradient is found for prevalence of musculoskeletal disorders [28], whereas the socioeconomic distribution of common mental disorders is less clear [29]. In the current study, the strong association between PCS and disability pension due to MSD may be influenced by the impact of manual work. Workers in lower-ranked occupations have less flexibility and control in the work situation and thereby less possibility to prevent MSD and subsequent sickness absence influenced by physical demands and poor ergonomic working environment [30]. In the current study, adjustment for education and occupational class attenuated the association between PSC and disability pension due to MSD, but a significantly excess risk persisted in quartiles 1 and 2. However, residual confounding by work-related factors cannot be ruled out [30].

Previous studies have reported that high BMI, smoking and physical inactivity predict disability pension due to low back pain and that an increase in BMI, physical inactivity and smoking increase the risk for disability pension due to MSD [21,31]. However, in the current study only smoking seemed to influence on the association between poor self-perceived physical health and disability pension. Lifestyle factors may be associated with self-perceived health [32]. The weak effect of lifestyle factors in the multivariate model may be due to this association.

### Strengths and limitations

The study had a prospective design with complete and accurate information of disability pension events from a National registry (FD-trygd). Self- perceived physical and mental health was measured with SF-12, a validated questionnaire that is widely used. A prior HUSK study found that individuals assessed their health more negatively before they were granted disability pension than afterwards [33]. To prevent misclassification of self-perceived health due to the long lasting process of being granted disability pension, we included only participants in paid work (at least 100 hours the last 12 months before baseline) and excluded all events of disability pension the first 12 months after baseline. Moreover, we tested the impact of extending the wash-out period to 24 months after baseline, finding only minor changes in the risk estimates. Nevertheless, the association between self-perceived physical health at baseline and disability pension may reflect the influence of already existing diseases. Osteoarthritis and rheumatoid arthritis aggravate with age and individuals with these conditions most likely have a higher risk of disability pension. However, the rates of disability pension across quartiles of PCS were stable during follow-up, indicating no particular peak in events for certain groups of PCS. Finally, the limited influence of competing risk outcomes in the association between self-perceived health and disability pension due to MSD, may strengthen the validity of our findings.

It has been reported that non-participants in HUSK had a higher risk for all-cause disability pension compared with participants [34]. However, corresponding differences in risk for disability pension due to MSD were minor and are not likely to affect the associations found in the current study. Further, the large group with missing values in SF-12 had a relatively high disability risk due to MSD; a finding that may reflect an association between reasons for not responding to items in the questionnaires and poor health.

Previous studies have found that women have a higher risk for disability pension and tend to develop musculoskeletal disorders earlier than men [35]. This was also found in the current study with a threefold increased disability risk for women. However, the relatively few events of disability pension among men (n = 47) restricted the possibility of gender specific analyses of the association between PCS and MCS, and subsequent disability pension. A previous HUSK study found that the association between PCS and all-cause disability pension was strong among both gender, whereas MCS was considerably weaker and inconsistent among women [36].

The relatively narrow age span in our study, 40–46 years at baseline and 47–52 at the end of follow up, may limit the generalizability to other age groups. However, the internal validity, i.e. the strong association between low PCS score and risk of disability pension due to MSD, is unlikely affected by the narrow age span.

## Conclusion

Middle-aged men and women in gainful work that identify physical health as a major limitation for performing ordinary work and domestic tasks, may have a relatively high likelihood of disability pension due to musculoskeletal diseases, whereas mental health seem to play a limited role.

## Competing interests

The authors declare that they have no competing interests.

## Authors’ contributions

All authors conceived of the study. IH and EHF drafted the manuscript and IH coordinated the process. All authors contributed to the design of the study and interpreted the data. EHF and IH made the statistical analyses. TR and GST advised the statistical analyses. All authors revised the manuscript for important content. All authors have read and approved the final version of the manuscript.

## Pre-publication history

The pre-publication history for this paper can be accessed here:

http://www.biomedcentral.com/1471-2458/14/167/prepub
